# Economic evaluation of a collaborative model of pharmaceutical care in an Irish hospital: cost-utility analysis

**DOI:** 10.12688/hrbopenres.13679.1

**Published:** 2023-03-21

**Authors:** Gráinne Kirwan, Aisling O'Leary, Cathal Walsh, Tamasine Grimes

**Affiliations:** 1 School of Pharmacy and Pharmaceutical Sciences, University of Dublin, Trinity College, Dublin, D02PN40, Ireland; 2 Pharmacy Department, Tallaght University Hospital, Tallaght, Dublin, D24, Ireland; 3 School of Pharmacy, The Royal College of Surgeons in Ireland, Dublin, D2, Ireland; 4 National Centre for Pharmacoeconomics, St James' Hospital, Dublin, D8, Ireland; 5 Health Research Institute and Department of Mathematics and Statistics, University of Limerick, Limerick, Ireland

**Keywords:** Hospital discharge, medication error, medication reconciliation, health economics, cost utility analysis, pharmaceutical care

## Abstract

**Background: **A complex, collaborative pharmaceutical care intervention including medication review and reconciliation demonstrated a statistically significant reduction in the prevalence of discharge medication error and improved quality of prescribing for hospitalised adults.  This study sought to assess the cost-effectiveness of this intervention.

**Methods: **A cost-utility analysis was undertaken using a decision-analytic framework. The evaluation was undertaken from the Health Service Executive’s perspective, the payer for primary and secondary care settings. Direct costs associated with managing hypothetical harm consequent to intercepted discharge medication error and consequences in terms of quality-adjusted life years loss were key input parameters. Analysis was structured within a decision tree model in Microsoft Excel® populated with consequences as utilities, estimated costs using macro- and micro-costing approaches, and event probabilities generated from the original study. Incremental analysis, one-way and probabilistic sensitivity analyses were performed.

**Results: **The results of analysis for the base-care demonstrated that the intervention dominated standard care with an incremental cost-effectiveness ratio of -€36,537.24/quality-adjusted life year, indicating that the intervention is less costly and more effective. The one-way and probabilistic sensitivity analyses both demonstrated that the intervention dominated standard care. The model was relatively robust to variation in input parameters through one-way sensitivity analysis. The cost of discharge medication error and effect parameters relating to standard care were most sensitive to change.

**Discussion: **The analysis demonstrated the cost-effectiveness of a complex pharmaceutical intervention which will support decision-making regarding implementation. This is the first cost-utility analysis of a complex, collaborative pharmaceutical care intervention, adding to the scant evidence-base in the field.

## Introduction

Hospitalised patients are at increased risk of medication misadventure relative to the general population, with a minority of medication errors leading to severe patient harm ^1, 2^. It is now well recognised that pharmaceutical care of hospitalised patients should include medication review and medication reconciliation at care transitions, facilitating integrated care within and between care settings ^3^. However, the economic value of such protective services to prevent medication error or mitigate the associated harm is uncertain ^4, 5^. Methodological variability is a feature of studies examining the prevalence and economic impact of medication error or the benefit of pharmaceutical care in hospitalised patients ^6, 7^. Furthermore, research to date tends to be undertaken within either the primary or secondary care setting, with relatively less work examining the transitional care journey ^6, 7^. 

A collaborative model of pharmaceutical care developed and examined in an Irish hospital through an uncontrolled before-after study, demonstrated a beneficial effect on discharge medication error rates and quality of prescribing ^8^. Medication reconciliation and medication review were integral component of this intervention. Evidence of its cost-effectiveness was required to support decision-making regarding implementation.

### Objectives

This study aimed to evaluate the cost-effectiveness of the intervention (Collaborative Ph Armaceutical Care at Tallaght Hospital, PACT) compared with standard care delivered to adult inpatients at an Irish university hospital, using a cost-utility analysis through a decision-analytic framework. 

Approval was obtained from the St James’s Hospital/Tallaght Hospital Joint Research Ethics Committee (SJH/AMNCH REC ref 2010/03/11).

## Methods

The economic evaluation was based on the primary study and the decision problem to be addressed was whether the intervention was more cost-effective than standard care (SC) ^8^. The evaluation was framed within the context of the study’s primary outcome measure, prevalence of discharge medication error.

The evaluation was undertaken from the perspective of the Health Service Executive (HSE), the payer for public healthcare in Ireland. The timeframe was one-year post-discharge. The weeks following hospital discharge have been identified as the highest risk for medication-related harm (MRH) ^4, 9^. However, a time horizon of one year was specified to consider manifestations of harm of longer latency or sequelae requiring lengthy remediation. As the time horizon did not exceed one year, discounting was not applied ^10^. The evaluation complied with the Consolidated Health Economic Evaluation Reporting Standards (CHEERS) and Irish national guidelines ^10– 12^. The intervention study was conducted between 2011 and 2012, published in 2014 and the cost-utility analysis study was undertaken between 2015 and 2017.

The setting is the Irish healthcare system. Study participants were adult inpatients acutely admitted to hospital who were taking at least three regular medicines and receiving medical, rather than surgical, care. There were no statistically significant differences in baseline population characteristics between the study groups in terms of age, gender, number of medicines, length of stay, or comorbidity burden ^8^.

The main differences between the intervention and comparator service have previously been reviewed and reported, and in brief, were as follows ^8^: PACT involved team-based working with pharmacist-led medication history taking and reconciliation on admission, pharmacist amending the prescription chart (collaborative prescribing) where necessary during admission; and pharmacist-led medication reconciliation at discharge, including discharge prescription amendments (collaborative prescribing) as required. Standard care (SC) comprised pharmacist admission medication history taking and documentation, and a routine inpatient clinical pharmacy service aligned with wards/clinical areas ( Table 1). SC pharmacists did not lead admission medication reconciliation, were not empowered to collaboratively prescribe, or amend the prescription and did not provide input at discharge.

**Table 1.** Comparison of intervention and standard care models (adapted from Grimes *et al.* 2014) ^10^.

	Standard care	Intervention
**Service alignment**	Aligned to a ward	Aligned to a medical team
**Clinical pharmacist involved**	Service delivered by routine clinical pharmacists	Service delivered by one of two intervention clinical pharmacists
**Pharmaceutical care delivered by pharmacist:**		
***At admission***	Contributed to admission medication history taking	Led admission medication history taking and reconciliation
***During admission***	Made minor changes and endorsements to the drug prescription and administration chart (drug chart), for example, clarify an intended formulation or notate to facilitate appropriate administration, for example, ‘before food’ Delivered routine clinical pharmacy tasks (drug chart review; therapeutic drug monitoring; medication review; contribution of suggestions to optimise medication use and medication information queries)	Made minor and major changes to the drug chart, as required, and these were co-signed by a medical practitioner Delivered routine clinical pharmacy tasks (drug chart review; therapeutic drug monitoring; medication review; contribution of suggestions to optimise medication use and medication information queries)
***At discharge***	No service at discharge	Discharge medication reconciliation Made minor and major changes to the discharge medication list, as required, and these were co-signed by a medical practitioner
**Pharmacist attributes**	Either basic grade or senior grade; some with postgraduate qualifications in hospital (clinical) pharmacy. No restriction in terms of postgraduate qualifications or years of experience applied.	Minimum three years post-registration experience with postgraduate qualification in hospital (clinical) pharmacy

### Model selection, structure, and assumptions

**Figure 1.**
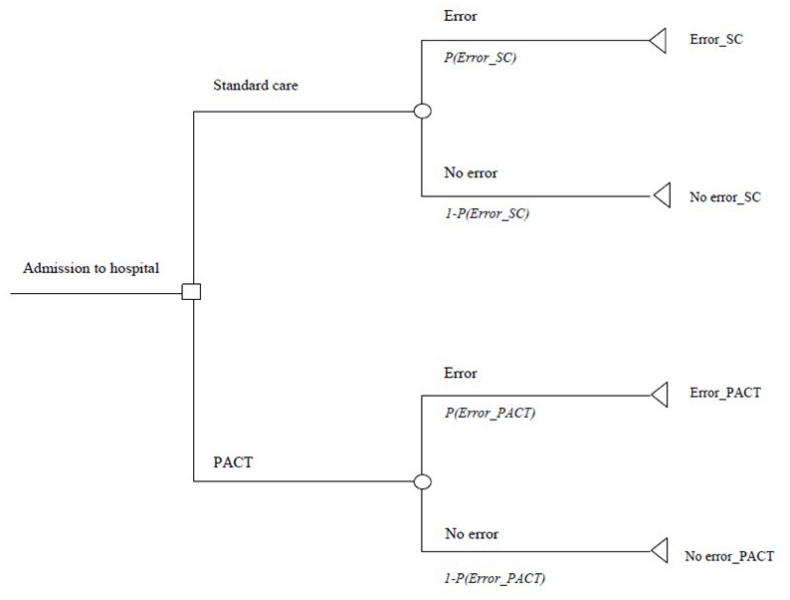
Decision tree model structure for the cost-effectiveness analysis of PACT versus Standard Care.

The evaluation was undertaken within a decision-analytic modelling framework. There was no interaction between individuals in the study on which the evaluation is based, while the patient pathways did not involve recurring events. The analysis was structured within a decision tree model built in Microsoft Excel® 2010 which describes the patient sequence from admission to discharge ( Figure 1).

The decision-analytic model reflected the study primary outcome measure: prevalence of discharge medication error. While budget allocations in Ireland for hospitals and general practitioners are administered by separate instruments of the HSE, the overall payer for the provision of healthcare to public patients in both settings is the HSE. The model assumes that the healthcare system, and budget allocation within, operates in cohesion, with no delineation between primary and secondary care.

All patients in the analysis were assumed to be entitled to full State endowed healthcare privileges and to not hold private health insurance and receive all aspects of pharmaceutical care, either PACT or SC. The accrual of costs due to post-discharge healthcare utilisation consequent to discharge medication error is reflected by the mean cost of error per patient in either group. The change in patient health-related quality of life (HrQoL) was identified as that caused by any discharge medication error(s) only. Therefore, patients who did not experience discharge medication error were assumed to have no change in their HrQoL.

### Evidence identification and synthesis

The decision model was populated with estimated costs and consequences in the form of utilities and event probabilities. Data generated from the primary study were used where possible, supplemented with additional data, where necessary ( Table 2). The probabilities of discharge medication error were taken directly from the original study as 65.3% and 13.9% of standard care and intervention group patients, respectively ^8^. The discharge medication errors identified during the intervention study were validated by two independent assessors, both clinical pharmacists, blinded to study allocation, and the level of agreement with the main investigator, measured using Cohen’s κ coefficient, was κ=0.8, indicating substantial agreement.

**Table 2.** Data sources, estimates and model input parameters of resource unit costs and quantities.

Resource type	Source of unit costs	Source of resource quantity	Derivation of cost	Costs	
				Standard care	Intervention
Pharmacist time	Department of Health salary scale	Time-and-motion study			
		Mean annual gross salary(42) (A)		48,960 *	64,489.5 ^†^
		Employer PRSI @ 10.75% of gross salary(13) (B)		5,263.2	6,932.621
		Imputed pension cost @ 13.10% of gross salary(13) (C)		6,413.76	8,448.125
		Overheads @ 40% of gross salary(13) (D)		19,584	25,795.8
		Total cost per annum (A+B+C+D)		80,220.96	105, 666
		Cost per hour of work ^‡^		55.00183	72.44773
		Cost per minute of work		0.9166972	1.2074622
Healthcare resource use post- discharge medication error		Extent and type of resource utilisation derived by expert elicitation ^13^			
GP consultation Prescription medication	HSE PCRS Statistical Analysis of Claims and Payments				
Hospital outpatient department visit/specialist referral Emergency department visit Hospital admission ICU admission	Ready Reckoner of Acute Hospital Inpatient and Daycase Activity and Costs 2013 ^21^				
**Model input parameter**					
Probability of discharge medication error	Primary study ^8^		Point estimate	0.653	0.139
Discharge medication error cost (€) ^&^	Expert elicitation ^13^		Mean (SD; 95%CI)	1091.12 (220.92; 702.51-1551)	638.73 (205.76; 248.41-1074.17)
Pharmacist cost (€) ^&^	Time-and-motion study		Mean (95%CI)	37.21 (29.18-47.39)	86.70 (58.84-119.77)
Utility decrement	Expert elicitation ^13^		Mean (SD)	-0.029 (0.072)	-0.023 (0.075)

* Mid-point of pay range (13) of the senior pharmacist salary scale (8 points) † Mid-point of pay range (13) of basic and senior pharmacist salary scales ‡ Calculation based on 37 hour working week (43), 24 days annual leave (assumption), 9 public holidays per annum (assumption), and one hour break per day (assumption) ^&^ Bootstrap mean costs and confidence intervals SD = standard deviation; 95%CI = 95% confidence interval

Identified costs included those associated with service delivery (labour costs) and those potentially incurred from consequences of discharge medication error. The sources of unit costs and resource quantities for each resource type included a mix of micro- and macro-costing, as described below.

Ascribing costs to potential discharge medication errors, had they not been intercepted, was challenged by the investigator’s ethical patient safety obligation and research ethics committee requirement to intervene where discharge medication errors may cause patient harm. Therefore, information on actual consequence of errors required to populate the model was not available and the cost of discharge medication error was derived through expert elicitation, which has previously been reported ^13^. In brief, using data from the parent intervention study, the hypothetical consequences of discharge medication error(s), in terms of diagnosis, healthcare resource utilisation and impact on health-related quality of life, were identified by expert judgement of anonymised cases. The experts comprised four practicing physicians, with 8-10 years’ experience in their fields of general practice (n=2) and hospital medicine (geriatrics n=1, nephrology n=1). Each expert was presented with a vignette describing the case for each patient who experienced discharge medication error(s) (n=81; n=66 standard care and n=15 intervention). The vignette contained anonymised demographics, medical history, presenting complaint, relevant clinical and laboratory findings, admission and discharge medication lists and a report of the discharge medication error(s). A total of 203 discharge medication errors were described within the 81 vignettes. Primary healthcare utilisation costs were derived from published tariffs, inpatient costs were derived by simulation in the hospital discharge activity database test environment and the difference between adjudicated baseline and post-error health state was expressed as quality-adjusted life year (QALY) decrement.

A time-and-motion study was conducted to derive units of pharmacist resource (time) required to deliver the intervention or comparator, with costs per unit of time subsequently derived from published salary scales ^14, 15^. These data had not been collected during the primary intervention study. The PACT model was reintroduced for a four-week period to facilitate data collection. One pharmacist and one medical team, both having participated in the primary study, were involved ^8^. Convenience sampling was used to enrol patients. To estimate the time required to deliver SC, patients receiving care from one of the four primary study physicians were randomly selected. The same patient inclusion and exclusion criteria as the primary study were applied. Overall time spent on clinical pharmacy activities per patient from admission to discharge was recorded. Independent observation was undertaken using a stopwatch as the measurement instrument. The Hawthorne effect was minimised by maintaining a distance between the observer and the pharmacist; concealing the stopwatch and data collection form; and avoiding conversation with the pharmacist ^16, 17^. Pharmacist’s time delivering care to enrolled patients from admission to discharge was recorded. Pay-related costs were calculated in accordance with guidelines using the appropriate mid-point on the published salary scales ( Table 2) ^14, 15^. Associated non-pay costs were incorporated in the calculation including employer’s Pay Related Social Insurance, pension, and overheads ^18^.

Quantities of healthcare resources utilised, as identified through expert elicitation, were weighted by the fixed monetary value of the unit cost estimates and summed to estimate total cost per patient ( Table 2) ^13^.

### Outcomes

QALY was the outcome of benefit measured, appropriate for a CUA ^10^. The baseline patient health-related quality of life (HrQoL), or the utility decrements associated with discharge medication error, were unknown. Utility values, pre- and post- discharge medication error, were derived through expert elicitation using the UK crosswalk value set to convert responses from the EQ-5D-5L instrument into utilities ^19^.

### Statistical analysis

Tests for differences between the study groups were undertaken in IBM SPSS Statistics®, Version 21 using Pearson’s χ ^2^ test for categorical data, the t-test for independent samples for parametric continuous data, and the Mann-Whitney U test for non-parametric data. Non-parametric bootstrapping with replacement involving 1000 replications was undertaken in Microsoft Excel, using an edited version of a published macro to obtain a 95% confidence interval (95%CI) for labour and discharge medication error cost estimates ^20^.

The payoffs, costs, and utilities, associated with the terminal node of each decision tree branch were entered. The expected value of the PACT base-case and the SC alternative were calculated by summing the products of the discharge medication error probability and consequences, in terms of costs and utilities ^20^. The incremental costs and outcomes were calculated, and the incremental cost-effectiveness ratio (ICER) was presented for the base-case, where:


$$\text{ICER=}\frac{{\text{Cost}}_{\text{PACT}}-{\text{Cost}}_{\text{SC}}}{{\text{QALY}}_{\text{PACT}}-{\text{QALY}}_{\text{SC}}}$$

The net monetary benefit (NMB) was calculated using the formula NMB = λ Effect - ∆ Cost, *i.e.,* NMB = λ (QALY _PACT_ – QALY _SC_) – (COST _PACT_ – COST _SC_), where λ represents the decision maker’s maximum willingness-to-pay [19]. The probability of the PACT intervention being cost-effective was represented through cost-effectiveness acceptability curves (CEACs). The threshold value for decision maker’s willingness-to-pay per unit change in outcome, or gain of one QALY, is unknown for this type of intervention, so a range of arbitrary ceiling ratios was employed, cognisant of the typical threshold for reimbursement of pharmaceutical products in Ireland being €45,000 per QALY ^22^.

One-way deterministic sensitivity analysis (DSA) of each of the model parameters was undertaken. The upper and lower bounds of the 95%CI for a given parameter were used where available; otherwise parameter values were varied by +/-20% ^10^. PSA was undertaken using Monte Carlo simulation to propagate uncertainty through the model. A total of 1000 iterations were performed. The beta distribution was applied to the probability parameters, while the gamma distribution, constrained on the interval from zero to positive infinity, was assigned to cost parameters, as costs are constrained to be non-negative and typically have a skewed distribution ^23^. Where utilities were negative, a transformation was made to disutilities, or utility decrements, and the gamma distribution was applied.

The PSA was undertaken in Microsoft Excel, utilising an edited version of a published macro to run 1000 Monte Carlo model simulations ^20^. The method of moments approach was used to estimate the hyperparameters, alpha and beta (α and β), of the beta distribution where α+β = [mean*(1-mean)/(SE ^2^)-1], α=mean* α+ β and SE is the standard error of the mean ^20^. The standard deviation (SD) was fixed at 0.2 for probability parameters in the absence of additional information. Similarly, the method of moments approach was used to estimate the hyperparameters, α and β, of the gamma distribution where α=mean ^2^/SE ^2^ and β=SE ^2^/mean ^20^.

## Results

Four experts provided judgement on 81 cases that involved one or more discharge medication error, as identified in the intervention study and reported in the expert elicitation study ^13, 24^. Of these, 75 were judged to have potential clinical consequences. Between 56 and 69 of the 81 cases were variably judged to require remedial healthcare utilisation. The mean calculated cost per case (representing an individual patient), based on all 81 cases, was €1009.58, 95% CI 726.64 to 1585.67. The mean QALY loss was 0.03 (95% CI 0.01 to 0.05). The bootstrap mean cost of discharge medication error per patient was calculated for PACT as €638.73 (95%CI 248.41-1074.17) and SC as €1091.12 (95%CI 702.51-1551). The mean (SD) utility decrement was -0.023 (SD 0.075) for PACT and -0.029 (SD 0.072) for SC.

Twenty-nine PACT patients and 63 SC patients were enrolled in the time-and-motion study, exclusion criteria were applied, and data were collected for 19 PACT patients and 30 SC patients. There were no significant differences between study groups in terms of patient gender, age, comorbidity burden, or number of admission medications ^13^. The mean pharmacist time spent per patient providing the PACT intervention was 72.8 (SD 52.47) and SC was 40.6 (SD 29.17) minutes.

An hourly difference in salary of approximately €17 between SC and PACT pharmacists was calculated, driven by differential labour costs between staff grades ( Table 2). Multiplying the cost per minute values obtained by the mean time to deliver the relevant pharmacy service resulted in a per patient cost of €87.91 (median €62.02; range €29.92-235.84) for PACT and €37.22 (median €32.08; range €13.75-159.51) for SC. The difference in mean cost between the groups was statistically significant (p = 0.002). Bootstrap mean costs were used in the base-case analysis ( Table 3). Bootstrapping identified the 95%CI for the mean cost of pharmacist time for PACT as €58.84-119.77 and for SC as €29.18-47.39.

**Table 3.** Base-case and one-way sensitivity analysis results.

Base-case					
Strategy	Cost (€)	Incremental cost (€)	Effect (QALY)	Incremental effect (QALY)	ICER (€/QALY)
SC	€749.71		-0.018978463		
PACT	€175.48	-€574.23	-0.003262228	0.015716235	-36, 537.24; Dominant
One-way sensitivity					
Parameter		Mean/point estimate (range)		Lower ICER value (€)	Upper ICER value (€)
***Probability of discharge medication error***		***Point estimate (+/-20%)***			
Standard care		0.653 (0.5224-0.7836)		-36217.1131; Dominant	-36732.82; Dominant
Intervention		0.139 (0.1112-0.1668)		-36165.69; Dominant	-36940.98; Dominant
***Discharge medication error cost (€)***		***Mean (95%CI)***			
Standard care		1091.12 (702.51-1551)		-20390.73; Dominant	-55644.98; Dominant
Intervention		638.73 (248.41-1074.17)		-39989.37; Dominant	-32686.06; Dominant
***Pharmacist cost (€)***		***Mean (95%CI)***			
Standard care		37.21 (29.18-47.39)		-36026.31; Dominant	-37184.98; Dominant
Intervention		86.70 (58.84-119.77)		-38309.93; Dominant	-34433.05; Dominant
***Utility decrement***		***Mean (+/-20%)***			
Standard care		-0.02906349626 (-0.0232508,-0.034876196)		-48171.29; Dominant	-29429.58; Dominant
Intervention		-0.023469269 (-0.01877542, -0.028163123)		-35080.89; Dominant	-38119.75; Dominant
Distributions and hyperparameters used in the PSA					
Parameter			Distribution		
***Probabilities***					
Discharge medication error, standard care			Beta (372.96, 198.19) * †		
Discharge medication error, PACT			Beta (44.78, 277.36) * †		
***Costs***					
Cost of discharge medication error SC			Gamma (24393.59, 0.04473) *		
Cost of discharge medication error PACT			Gamma (9636.58, 0.06628) *		
Cost of pharmacist SC			Gamma (59903.58, 0.00062) *		
Cost of pharmacist PACT			Gamma (30197.42, 0.00287) *		
***Utilities***					
Decrement SC			1-(Gamma) (13279.82, 0.00008) *		
Decrement PACT			1-(Gamma) (2828.56, 0.00036) *		

*Alpha and beta hyperparameters of the beta and gamma distributions in parentheses †SD fixed at 0.2 during estimation of SE

The expected values for costs and utilities were identified for PACT and SC, from which the incremental costs and effects were calculated. The ICER for PACT relative to SC in the base-case was negative (-€36, 537.24/QALY) indicating that the PACT intervention dominates SC, i.e., cost-effective.

**Figure 2.**
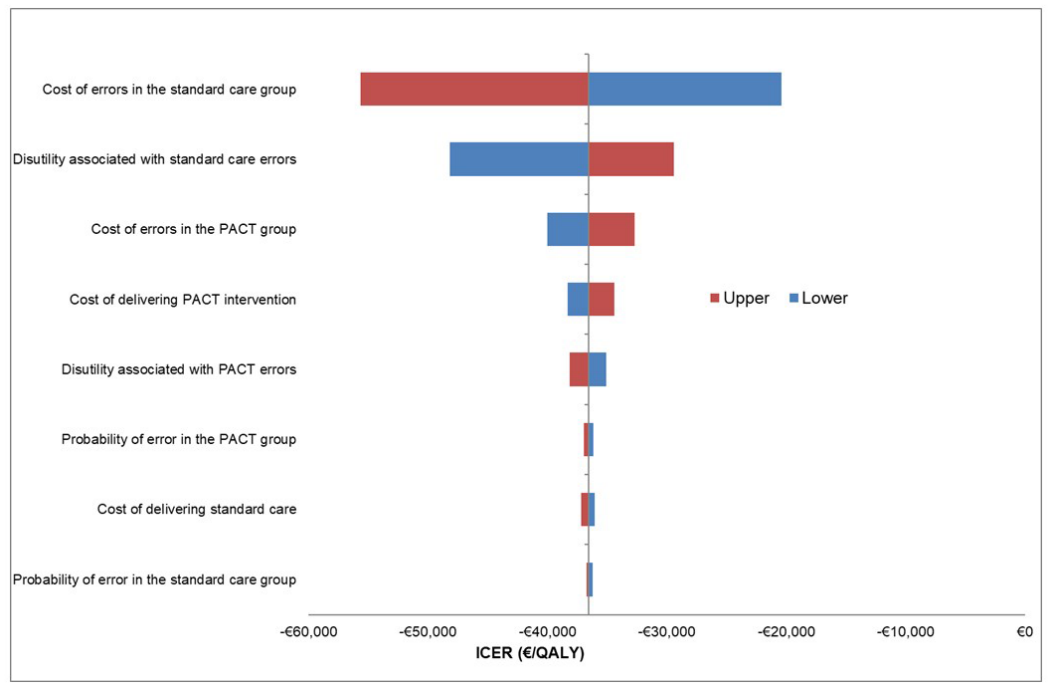
Tornado diagram of one-way sensitivity analysis. PACT = Collaborative PhArmaceutical Care at Tallaght Hospital (the intervention); SC = Standard care.

**Figure 3.**
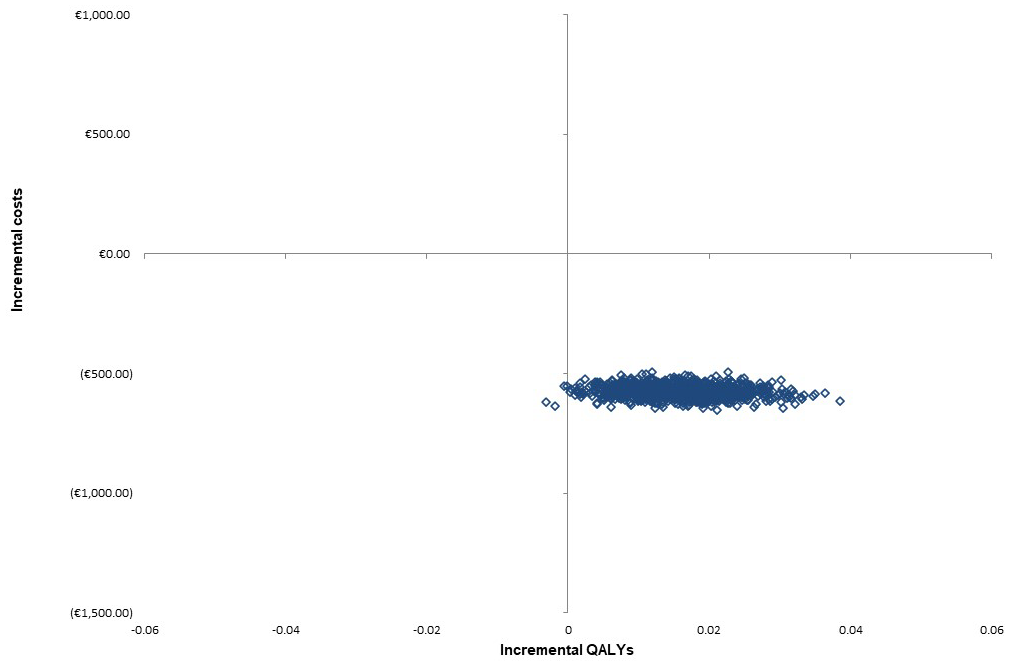
Scatter plot of the incremental cost/QALY pairs of PACT vs SC. PACT = Collaborative Pharmaceutical Care at Tallaght Hospital (the intervention), QALY = Quality Adjusted Life Years, SC = Standard Care.

**Figure 4.**
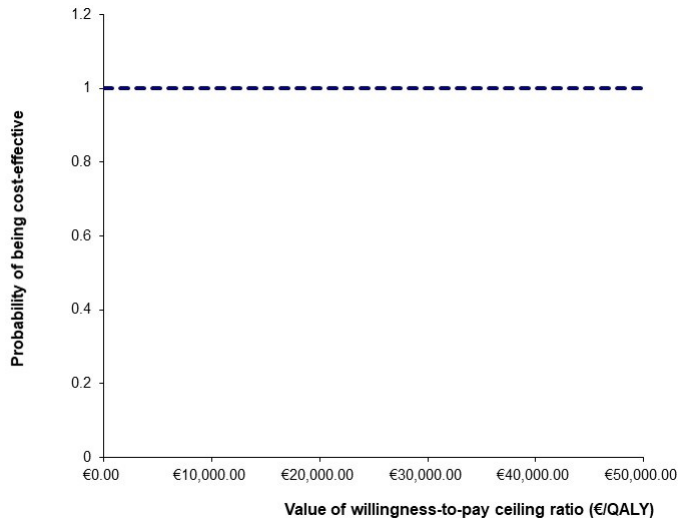
Cost-effectiveness Acceptability Curve.

The DSA showed that the model was robust to variation in individual parameters ( Figure 2); all deterministic ICERs indicated that PACT dominated SC. The parameters most sensitive to variation were the cost of SC discharge medication errors and the QALY decrement for SC. The distributions and their associated hyperparameters for each input within the PSA are summarised ( Table 3). The PSA-derived cost-effectiveness plane for PACT versus SC produced illustrates that cost-effect pairs generated are mostly located in the south-east quadrant, confirming that PACT dominates SC, and is cost-effective ( Figure 3). A CEAC illustrated the probability of cost-effectiveness of PACT at several ceiling willingness-to-pay values ( Figure 4). PACT was certain to be cost-effective (probability of 1) across all cost-effectiveness threshold values from €0-50,000. The intervention NMB was positive for each PSA produced incremental cost and effect pairing, even when the cost-effectiveness threshold was set at zero. Therefore, this is a cost saving intervention.

## Discussion

The key study findings are that a complex, multi-faceted, collaborative pharmaceutical care intervention demonstrated cost-effectiveness compared to standard ward-based clinical pharmacy care. The sensitivity analyses confirmed that PACT dominated SC. The model was relatively robust to variation in input parameters through one-way sensitivity analysis; however, it was noted that the cost of discharge medication error and effect parameters relating to SC were most sensitive to change. This study is the first CUA of a complex pharmaceutical care intervention that includes medication reconciliation and medication review, delivered by hospital pharmacists in a collaborative pharmaceutical care model in Ireland and adds to the scant cost-effectiveness evidence available in the field.

A strength of this study was the use of national, rather than hospital specific, data to estimate labour costs and the economic burden of post-discharge management of discharge medication errors, potentially extending generalisability to similar national contexts. However, the intervention, PACT, was developed in the study hospital, and the economic findings are specific to the investigated intervention and standard care models. Given the considerable practice variation in the delivery of clinical pharmacy services in Ireland, the nature of the comparator is likely to vary widely between hospitals, with implications for the generalisability and likelihood of enhanced cost effectiveness in hospitals with less intensive service ^25^. Variation between pharmacists delivering the intervention due to differences in training and experience may also influence outcomes. Consideration should therefore be given to post-registration professional development to harmonise and credential hospital pharmacist competence ^26^.

The assumptions made in structuring the model, and the sources of data used to populate it, result in several potential limitations. While the analysis was based on a published study, it was not a trial-based economic evaluation, as direct costs associated with patient care were not available and were established by expert elicitation. It was not possible to capture inter-personal variation in the time-and-motion study of the simulated PACT service as only one pharmacist was involved, a limitation addressed through sensitivity analysis. The assumption that all patients were entitled to full statutory healthcare privileges meant that potential cost offsets due to patient co-payments or health insurance contributions were not captured, limiting generalisability beyond the specific state-assisted cohort. Other costs not included in the analysis include: litigation costs; opportunity costs arising from time spent by healthcare professionals resolving discharge medication error; the impact on medical/surgical or nursing service costs ^27^. It is speculated that the PACT model likely resulted in a net benefit in terms of physician/surgeon time, however, the model may have had a deleterious impact on nursing time ^28^.

The results are comparable to economic analyses of broadly similar interventions undertaken in Sweden, the United Kingdom and United States (US), all demonstrating the net economic benefit of pharmacist-led interventions with a medication reconciliation component ^4, 29, 30^. The US study identified a further potential economic benefit by targeting the discharge medication reconciliation service to patients at increased risk of medication-related rehospitalisation ^4^. 

The findings from our time-and-motion study provide an estimate of the time required to deliver end-to-end pharmaceutical care, inclusive of elements at care interfaces and during the inpatient care period. This information will be useful to policy makers and managers planning service delivery and future cost-effectiveness analyses. It is difficult to directly compare our findings with previous studies, given that most reported estimates assess the time for admission or discharge medication reconciliation delivery, or both, without other pharmaceutical care service elements ^31– 33^. The combined evidence suggests that medication reconciliation consumes most of the pharmacist’s time delivering end-to-end hospital inpatient care and that investment in admission medication reconciliation complements and reduces the time required to perform the process well at discharge.

A significant proportion of healthcare resource utilisation consequent to discharge medication error (which often derives from admission medication error) is likely to occur post-discharge in primary care ^34, 35^. Although the costs associated with outpatient resource use are relatively small compared with the inpatient costs, in the context of large volumes of patient discharge activity and opportunity costs, the burden of remediating consequences of discharge medication error may be substantial. The data which informed the value of the study model parameters considered this, as did the study perspective of the health service provider being both the payer and the monetary beneficiary of the intervention. This approach was employed previously and concords with Irish policy to achieve integrated care ^4, 36^. 

The paucity of good quality data on the manifestations of harm consequent to discharge medication error has been noted elsewhere as a reason for diminished certainty in CUA findings ^37^. The Expected Value of Perfect Information approach may support identifying the upper bound on the value of undertaking additional research to reduce uncertainty in decision-making ^38^. Although this study established the cost-effectiveness of PACT, a Budget Impact Analysis would forecast the impact of intervention adoption and diffusion within the Irish health service context ^39^.

## Conclusion

This CUA demonstrates that the PACT intervention is cost saving and cost-effective compared to standard care, notwithstanding that confidence in the results is diminished by the lack of data on the actual costs and health impact of discharge medication errors. The ICERs generated indicate that the PACT intervention dominated standard care, and sensitivity analyses assured the results were robust to parameter variation. The findings may support decision making regarding PACT implementation and contribute to the economic evidence-base regarding pharmaceutical care, collaborative models of care and medication reconciliation-based interventions.

## Data Availability

Zenodo: PACT Cost utility assessment data. https://doi.org/10.5281/zenodo.7684239 ^24^ This project contains the following underlying data: Bootstrapping of error costs.xls Bootstrapping of time costs.xls CEAC 2022.xlsm Model.xlsx PACT arm_time and motion.xlsx PSA.xls Standard care arm_time and motion.xlsx Zenodo: Economic evaluation of a collaborative model of pharmaceutical care in an Irish hospital: Cost-utility analysis. Consolidated Health Economic Evaluation Reporting Standards (CHEERS) checklist. https://doi.org/10.5281/zenodo.7684563 ^12^ This project contains the following extended data: CHEERS checklist 2023-02-28.pdf Data are available under the terms of the Creative Commons Attribution 4.0 International license (CC-BY 4.0).
