# Supplementary material for: Economic evaluation of a collaborative model of pharmaceutical care in an Irish hospital: cost-utility analysis
Source: HRB Open Res. 2023 Mar 21;6:19. [Version 1] doi: 10.12688/hrbopenres.13679.1 (PMC10382783; doi:10.12688/hrbopenres.13679.1)
Supplement: Peer review history

# Peer review: Reviewer response for version 1

**DOI:** 10.21956/hrbopenres.14960.r34020

**Published:** 2023-07-28

**Authors:** Alemayehu Mekonnen

**Affiliations:** 1 Deakin University, Burwood, Australia

Overall, the manuscript reads very well, and I applaud the effort the authors have done in completing a manuscript of interest for medication safety researchers/policy makers. The manuscript adds important findings to the scant cost-effectiveness evidence available in the international literature and it is novel in that it was the first cost-utility analysis of a complex pharmaceutical care intervention that includes medication review and reconciliation. 

 I have only minor comments to the manuscript, as below:

 It looks like the discharge medication error cost considers costs related to harm diagnosis, healthcare utlisation and HrQoL. As there is little information on the type of discharge errors, would the authors also consider additional costs incurred due to commission errors? It would be helpful, maybe as appendix, to add some examples of the discharge errors that were deemed consequential and were added to the CUA model.

Is the work clearly and accurately presented and does it cite the current literature?

Yes

If applicable, is the statistical analysis and its interpretation appropriate?

I cannot comment. A qualified statistician is required.

Are all the source data underlying the results available to ensure full reproducibility?

Yes

Is the study design appropriate and is the work technically sound?

Yes

Are the conclusions drawn adequately supported by the results?

Yes

Are sufficient details of methods and analysis provided to allow replication by others?

Yes

Reviewer Expertise:

Medication safety

I confirm that I have read this submission and believe that I have an appropriate level of expertise to confirm that it is of an acceptable scientific standard.

# Peer review: Reviewer response for version 1

**DOI:** 10.21956/hrbopenres.14960.r33632

**Published:** 2023-05-09

**Authors:** Jonathan Karnon

**Affiliations:** 1 College of Medicine and Public Health, Flinders University, Adelaide, SA, Australia

The manuscript reports an impressive cost utility analysis of a collaborative model of pharmaceutical care for adult inpatients taking at least three regular medicines and receiving medical care.

 It would be useful to clarify the timing of the medication errors represented in the model structure. The text refers only discharge medication errors, so the model only reflects discharge errors? If so, some commentary on the likelihood and potential impact of an intervention effect on pre-discharge errors would be useful.

 The described elicitation process is impressive, but further details (maybe in an appendix) would be helpful, e.g., were individual estimates from the 4 physicians synthesised? How long did the elicitation process take (81 vignettes is a lot)? How were the experts asked to define baseline and post-error health states? Was it possible to compare predicted effects for similar vignettes (as a form of validation)?

 The following explanation of inpatient costing is not very informative: “inpatient costs were derived by simulation in the hospital discharge activity database test environment”

 The described time and motion study to estimate intervention costs is also impressive, though the exclusion of 10 of 29 PACT patients from the time and motion study was unexpected – why were so many excluded? Does this mean PACT pharmacists were spending 1/3 of their time providing services to non-eligible patients – what impact might this have on the cost-effectiveness of the intervention?

 In Table 2, could you present confidence intervals around the probs of discharge medication errors?

 The described model analysis is good, but for which model parameters were 95% confidence intervals not available? I would suggest threshold analyses might be a useful addition, e.g., what differences in key parameters would be required for the intervention to not be cost-effective?

 My preference is not to present negative ICERs – they cannot be interpreted, but rather to say the intervention was dominant. Table 3 is a little confusing – my preference would be not to present negative ICERs and to state which option was dominant, e.g., was SC dominant when applying the lower medication error cost estimate?

 In the discussion it would be useful to comment on the availability of any primary studies that have investigated the costs and outcome effects of medication errors at discharge. Have any studies tried to collect primary data to estimate such effects? E.g., from https://www.uptodate.com/contents/hospital-discharge-and-readmission, “A 2023 meta-analysis of 25 randomized trials of over 15,000 hospitalized adults, followed from 1 to 20 months, demonstrated that medication review was likely to reduces the risk of hospital readmissions [16].” How do these findings relate to the elicited effects?

Is the work clearly and accurately presented and does it cite the current literature?

Yes

If applicable, is the statistical analysis and its interpretation appropriate?

Yes

Are all the source data underlying the results available to ensure full reproducibility?

Partly

Is the study design appropriate and is the work technically sound?

Yes

Are the conclusions drawn adequately supported by the results?

Yes

Are sufficient details of methods and analysis provided to allow replication by others?

Partly

Reviewer Expertise:

Economic evaluation

I confirm that I have read this submission and believe that I have an appropriate level of expertise to confirm that it is of an acceptable scientific standard.
